# Synthesis and Properties of a Selective Inhibitor of Homeodomain–Interacting Protein Kinase 2 (HIPK2)

**DOI:** 10.1371/journal.pone.0089176

**Published:** 2014-02-24

**Authors:** Giorgio Cozza, Sofia Zanin, Renate Determann, Maria Ruzzene, Conrad Kunick, Lorenzo A. Pinna

**Affiliations:** 1 Department of Biomedical Sciences, University of Padova, and CNR Institute of Neurosciences, Padova, Italy; 2 Technische Universität Braunschweig, Institut für Medizinische und Pharmazeutische Chemie, Braunschweig, Germany; 3 Venetian Institute of Molecular Medicine (VIMM), Padova, Italy; Cedars-Sinai Medical Center; UCLA School of Medicine, United States of America

## Abstract

Homeodomain-interacting protein kinase 2 (HIPK2) is a Ser/Thr kinase controlling cell proliferation and survival, whose investigation has been hampered by the lack of specific inhibitors able to dissect its cellular functions. SB203580, a p38 MAP kinase inhibitor, has been used as a tool to inhibit HIPK2 in cells, but here we show that its efficacy as HIPK2 inhibitor is negligible (IC_50_>40 µM). In contrast by altering the scaffold of the promiscuous CK2 inhibitor TBI a new class of HIPK2 inhibitors has been generated. One of these, TBID, displays toward HIPK2 unprecedented efficacy (IC_50_ = 0.33 µM) and selectivity (Gini coefficient 0.592 out of a panel of 76 kinases). The two other members of the HIPK family, HIPK1 and HIPK3, are also inhibited by TBID albeit less efficiently than HIPK2. The mode of action of TBID is competitive with respect to ATP, consistent with modelling. We also provide evidence that TBID is cell permeable by showing that HIPK2 activity is reduced in cells treated with TBID, although with an IC_50_ two orders of magnitude higher (about 50 µM) than in vitro.

## Introduction

The CMGC group of the human kinome is split into several branches, one of which, also including DYRKs and CLKs, gives rise to a sub-branch composed by so called “homeodomain-interacting protein kinases” (HIPKs). Four HIPKs are present in human, with HIPK2 attracting special attention for its role as a regulator of growth and apoptosis in various types of cells [1]. HIPK1/2 double deficient mice exhibit defects in hematopoiesis, vasculogenesis and angiogenesis [2]. HIPK2 was firstly recognized as a DNA damage responsive kinase exerting a tumor suppressor function by mediating p53 activation [3], [4]. HIPK2 however can also mediate apoptosis in the absence of p53 [5], [6], [7] and a number of observations summarized in [1] strongly argue for additional non apoptotic roles of HIPK2, whose precise understanding will require the identification of new HIPK2 targets.

These studies have been hampered by the lack of selective inhibitors of HIPK2. Small cell permeable inhibitors of protein kinases have become invaluable reagents for dissecting signaling pathways mediated by each of them. In recent years a huge repertoire of compounds purported to be “specific” toward a large number of protein kinases have become available. Since however the human kinome is composed by some 500 members the issue of selectivity is critical and only in a limited number of cases inhibitors have been shown to display a really narrow selectivity window hitting only few and in very rare cases one individual protein kinases [8], [9].

In the case of HIPK2 the rational design of specific inhibitors has never been reported, the only HIPK2 inhibitor mentioned in the literature being SB203580, a compound firstly employed as HIPK2 inhibitor [10] because this kinase displays features similar to p38 like MAP kinase, whose susceptibility to SB203580 was already established. Consequently several laboratories exploited SB203580 as a “HIPK2 inhibitor” (e.g. [11], [12]), based on the assumption that its targeting of HIPK2 is selective. However by profiling SB203580 on a panel of 71 protein kinases at 1 µM concentration, inhibition of HIPK2 was negligible (14%) as compared to that of 6 protein kinases which were inhibited >60%, and it remained below the average inhibition of the whole panel (17.7%) [9]. Moreover the members of the HIPK family are not among the kinases inhibited by SB203580 (nor by any other compound examined) in a comprehensive profiling of kinase inhibitors selectivity [13]. This sheds doubts on the interpretation of the effects of SB203580 as really mediated by cellular HIPK2 blockage.

In the course of our studies aimed at the identification and development of compounds able to inhibit CK2, a highly pleiotropic kinase [14], [15], playing a key role as an anti-apoptotic agent [16] and whose abnormally high level enhances the tumor phenotype through a non oncogene addiction mechanism [17], [18], we observed that several potent CK2 inhibitors also exert a drastic effect on a few other protein kinases, notably DYRK1A, PIMs and HIPK2 [19], [20]. This was especially true of the most common CK2 inhibitors, TBB (TBBt) and TBI (TBBz) and of related tetrabromo-benzimidazole derivatives.

These observations prompted us to design modifications of the tetrabromo-benzimidazole scaffold aimed at decreasing the efficacy toward CK2 and other kinases drastically inhibited by TBI and TBB, while maintaining or eventually improving that toward HIPK2.

Here we describe the properties of one of these derivatives, 4,5,6,7-tetrabromo-2-(1*H*-imidazol-2-yl)isoindoline-1,3-dione (TBID) which is able to inhibit HIPK-2 with a selectivity much higher than that of TBI, not to say of SB203580, whose ability to inhibit HIPK2 is in our hands negligible. These properties in conjunction with cell permeability, make TBID the first choice inhibitor of HIPK2 presently available for both in vitro and in cell studies.

## Materials and Methods

### Chemistry

Synthesis and details concerning compounds **5a-5i** are provided in Supporting Information.

Instruments were used and procedures for compound characterization were carried out as published before [21], [22].

### Source and Purification of Protein Kinases

Native CK2 and CK1 were purified from rat liver [23].

The source of HIPK2 and of all of the other protein kinases used for specificity assays is as described elsewhere [9].

### Kinase Inhibition Assays

HIPK2 (63 ng) was preincubated at 37°C for 10 minutes either in the absence or in the presence of increasing amounts of each inhibitor in a final volume of 20 µl of solution containing 50 mM Tris-HCl pH 7.5, 0.1% (v/v) 2-mercaptoethanol, 0.1 mM EGTA, 10 mM magnesium acetate. The reaction was started by addition of 5 µl of a reaction mixture containing 20 µM [^33^P-ATP] (500–1000 cpm/pmol), and the synthetic peptide substrate NKRRRSPTPPE [24] (600 µM, unless differently indicated). Similar results were obtained by replacing the peptide with MBP (0.33 mg/ml). The reaction was stopped by addition of 5 µl of 0.5 M orthophosphoric acid before spotting aliquots onto phosphocellulose filters. Filters were washed in 75 mM phosphoric acid (5–10 ml/each) four times and then once in methanol and dried before counting.

PIM1, CK2, CK1 phosphorylation assays were performed following similar procedures used for HIPK2 except that preincubation was omitted. In detail, PIM1 activity was determined at 100 µM ATP concentration and in the presence of 30 µM synthetic peptide substrate RKRRQTSMTD. CK2 and CK1 activities were assayed in a buffer containing 50 mM Tris/HCl (pH 7.5), 100 mM NaCl, 12 mM MgCl_2_, 100 µM specific peptide substrate RRRADDSDDDDD (for CK2), or 200 µM IGDDDDAYSITA (for CK1) and 20 µM [*γ*-^33^P]ATP (500–1000 c.p.m./pmol).

Conditions for the activity assays of all other protein kinases tested in selectivity experiments are as described or referenced in [9].

### Kinetics

Initial velocities were determined at each of the ATP concentration tested, at 1 mM NKRRRSPTPPE peptide concentration. Km values were calculated either in the absence or in the presence of increasing concentrations of inhibitor, from Lineweaver-Burk double-reciprocal plots of the data. Inhibition constants were then calculated by linear regression analysis of Km/Vmax versus inhibitor concentration plots.

### Selectivity Parameters

Lorenz curves were derived from the selectivity data. Gini coefficients and hit rates (expressing the percent of kinases inhibited >50% by a given compound) were calculated as described in [25].

### Molecular Modeling

#### Model building

Human HIPK2 protein kinase was built using an homology modeling approach implemented into Molecular Operating Environment (MOE) [26] with DYRK1A as template (PDB code: 2WO6) [27]. All the ligands and cofactors were removed; hydrogen atoms were added using standard geometries to the protein structure with the MOE program. To minimize contacts between hydrogen’s, the structures were subjected to Amber99 force field minimization until the *rms* of conjugate gradient was <0.05 kcal mol^−1 ^Å^−1^ keeping all the heavy atoms fixed [26]. To strictly validate the model generated and to calibrate our high-throughput docking protocol, a small database of known HIPK2 inhibitors was built and a set of docking runs was performed.

#### Molecular docking

After the calibration phase, all compound structures were docked directly into the ATP binding site of the human HIPK2 model, by using the docking tool part of the GOLD suite [28]. Searching was conducted within a user-specified docking sphere (12 Å from the center of the binding cleft), using the Genetic Algorithm protocol and the GoldScore scoring function. GOLD performs a user-specified number of independent docking runs (50 in our specific case) and writes the resulting conformations and their energies in a molecular database file. Prediction of small molecule-enzyme complex stability (in terms of corresponding pKi value) and the quantitative analysis for non-bonded intermolecular interactions (H-bonds, transition metal, water bridges, hydrophobic, electrostatic) were calculated and visualized using several tools implemented in MOE suite.

### Cell Culture and Treatment

HepG2 cells (human hepatocellular carcinoma) were cultured in Dulbecco's modified Eagle's medium (DMEM; Sigma) supplemented with 10% fetal calf serum, 2 mM L-glutamine, 100 unit/ml penicillin and 100 µg/ml streptomycin; CEM cells (human T lymphoblastoid cells) were cultured n RPMI-1640 (Sigma) with the same supplements. Cells were cultured in an atmosphere containing 5% CO_2_, at 37°C. Treatments with TBID were performed in the same medium but with 1% fetal calf serum; control cells were treated with the solvent (DMSO). Total cell lysates were prepared as in [29].

### Cellular HIPK2 Kinase Assays

Endogenous HIPK2 activity was evaluated by measuring the phosphorylation level of its target site Ser46 of p53: to this purpose, CEM cells were treated for 6 h as indicated, then lysed. 10 µg of total proteins were loaded on 11% SDS-PAGE, blotted on Immobilon-P membranes (Millipore), and analyzed by western blot (WB) using an anti-phospho Ser46 p53 antibody (BD Biosciences); chemiluminescence signals were acquired with a Kodak 4000MM Pro Image Station. Bands were quantified by Carestream Molecular Imaging Software (Kodak) and the obtained values were normalized to total p53 signal with a Cell Signaling Technology antibody; anti-actin (Sigma) was used as loading control.

Alternatively, HIPK2 was immunoprecipitated with 2.5 µl anti-HIPK2 (Epitomics) from 350 µg of total lysate proteins deriving from HepG2 cells either treated or not with TBID following a protocol elsewhere described [30]. An aspecific antibody was used as negative control. Immunoprecipitated HIPK2 activity was measured towards the specific peptide substrate (NKRRRSPTPPE) at 1.6 mM concentration, for 10 min at 30°C, under the same conditions described above for the in vitro kinase assay. Peptide radioactivity was measured after sample spotting on phospho-cellulose paper, washing and scintillation counting, as in [30], while the amount of HIPK2 immunoprecipitated was evaluated by WB.

### Cell Viability and Apoptosis Assays

Cell viability was evaluated by means of MTT (3-(4,5-dimethylthiazol-2-yl)-3,5-diphenyltriazolium bromide) reagent; 10^5^ cells/100 µl were incubated in a 96-well plate and treated for 6 h as indicated. 1 h before the end of the incubation, 10 µl of MTT solution (5 mg/ml in PBS) was added to each well. Incubations were stopped by addition of 20 µl of lysis solution at pH 4.7, as described elsewhere [30]. Plates were read for OD at λ 590 nm, in a Titertek Multiskan Plus plate reader (Flow Laboratories). Apoptosis was evaluated looking for the cleavage of the caspase substrate PARP with anti-PARP antibody (Roche), recognizing both the full length (116 kDa) and the cleaved fragment (85 kDa) of PARP.

## Results and Discussion

### 1. Synthesis of Tetrabromoisoindoline-1,3-dione Derivatives that Inhibit HIPK2

**Figure 1:**
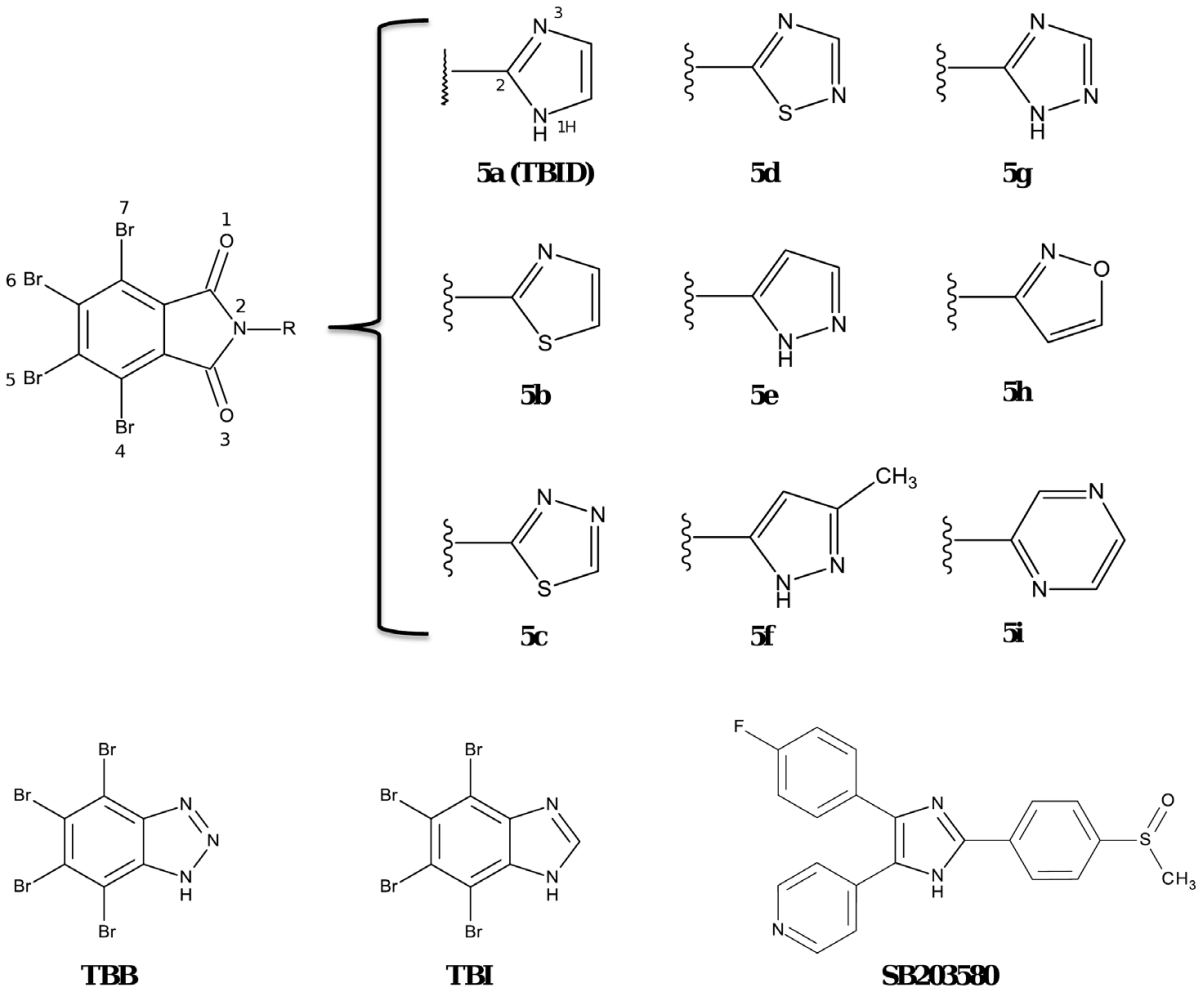
Structures of 2-aryl-4,5,6,7-tetrabromoisoindoline-1,3-dione derivatives. The 2-aryl-4,5,6,7-tetrabromoisoindoline-1,3-dione scaffold is shown on the left, in the upper part of the figure, where R was variably replaced in the individual compounds, as indicated on the right. In the lower part of the figure the formulae of TBB, TBI and SB203580 are reported for comparison.

The structures of two commonly used CK2 inhibitors, TBB and TBI are shown in Figure 1. These compounds share a number of bromine atoms clustered on their benzene ring which are essential for interaction with the kinase active site [31], [32]; they also share the ability to inhibit HIPK2 besides CK2. This is especially true of TBI whose IC_50_ values with either HIPK and CK2 are nearly identical (0.7 vs 0.6 µM, [19] and Table 1). Since these compounds have been shown to become entrapped in a hydrophobic cavity adjacent to the ATP binding site, whose size in CK2 is particularly small, owing to a number of bulky side chains which are replaced by smaller ones in the majority of protein kinases, HIPK2 included, we reasoned that a device to reduce affinity toward CK2 as compared to that toward HIPK2 could be to increase the size of the tetrabrominated ligand. This goal was attained by derivatizing a tetrabromoisoindoline-1,3-dione scaffold (nearly super-imposable to that of TBI) with an imidazole group, to give rise to 4,5,6,7-tetrabromo-2-(1*H*-imidazol-2-yl)isoindoline-1,3-dione (TBID, **5a**), The synthesis of TBID (**5a**) and analogs **5b**-**5i** was carried out following a published general synthesis protocol [33] by reacting tetrabromophthalic anhydride with suitable aminosubstituted hetarenes (File S1).

**Table 1:** IC_50_ (µM) of 2-aryl-4,5,6,7-tetrabromoisoindoline-1,3-dione derivatives (Figure 1) for HIPK2, CK2, PIM1, CK1; TBB and TBI values are drawn from [19].

Entry	HIPK2	CK2	PIM1	CK1
TBID (**5a**)	0.33	5.50	>40.0	>40.0
**5b**	0.72	16.0	40.0	9.3
**5c**	2.6	24.0	>40.0	>40.0
**5d**	2.5	35.0	>40.0	>40.0
**5e**	>40.0	23.0	>40.0	>40.0
**5f**	>40.0	>40.0	>40.0	>40.0
**5g**	22.7	25.0	>40.0	>40.0
**5h**	23.6	>40.0	>40.0	>40.0
**5i**	3.62	>40.0	>40.0	>40.0
**TBB**	5.3	0.15	1.04	>40.0
**TBI**	0.7	0.60	0.115	15
**SB203580**	>40.0	n.d.	n.d.	n.d.

n.d. = not determined.

As shown in Table 1, TBID (**5a**) inhibits HIPK2 with the same efficiency as TBI, while displaying toward CK2 a more than10-fold higher IC_50_ value.

Compared to TBID, all analogs **5b-5i** were less potent HIPK2 inhibitors (Table 1, Figure 1). The outcome of this analysis underscores the crucial role of the 5-membered imidazole ring to achieve high inhibitory efficiency toward HIPK2: in fact its replacement with 6-membered aromatic rings or even with 5-membered rings with different spacing between the nitrogen atoms promotes big rises in IC_50_ values. Especially telling in this respect is the loss of inhibitory efficacy underwent by compound **5e**, whereas compounds **5c** and **5d**, albeit less potently than TBID, still inhibit significantly HIPK2, highlighting the crucial role of a basic nitrogen atom at position 3.

**Figure 2:**
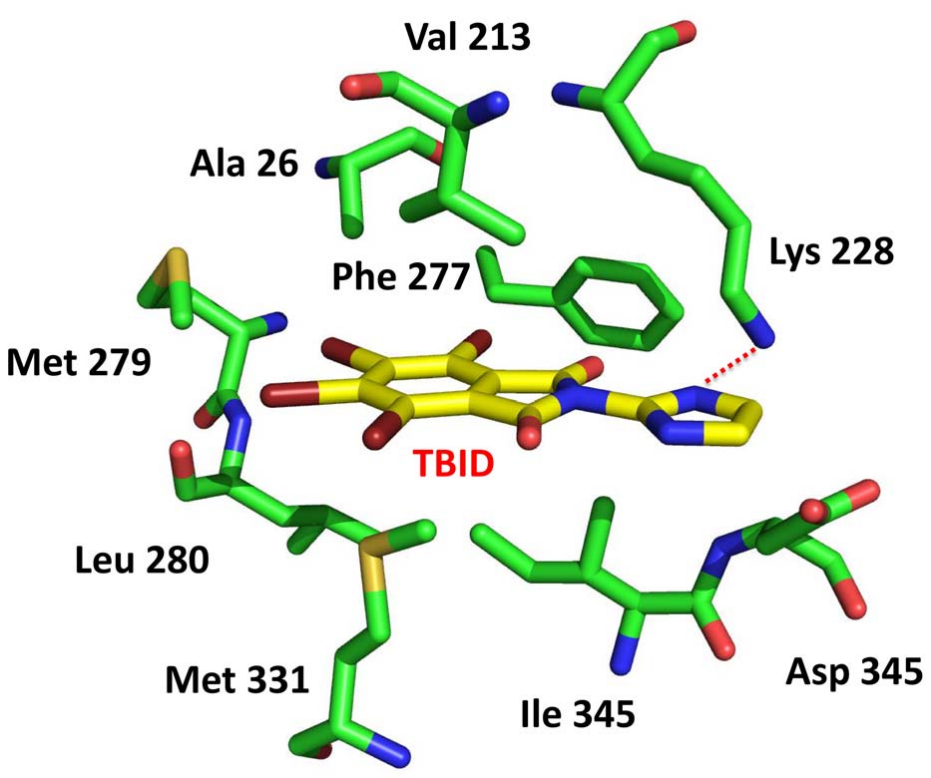
In silico analysis of HIPK2-TBID complex. Molecular docking of TBID (yellow) was performed in the active site of the human HIPK2 model (green).

Modeling accounts for the above observations disclosing the role of this nitrogen for binding. HIPK2 models were built and prepared as described in the experimental section; after a docking calibration phase, a small database of tetrabromo phthalimides was built and a series of docking experiments were performed. As shown in Figure 2 TBID interacts with the hinge region through hydrophobic interactions between Val 213, Val 261, Phe 277, Leu 280, Met 331, Ile 345, and the tetrabromine moiety, while the symmetric nitrogen atom at position 3 interacts with the catalytic Lys 228, thus playing a crucial role in the binding architecture. In fact compounds **5b**, **5c**, **5d**, **5g**, **5h** and **5i** still inhibit HIPK2 in the low micromolar range establishing the same electrostatic interaction found in the case of TBID. By contrast **5e**, a TBID isomer, presenting a pyrazol ring instead of the imidazole of TBID cannot perform the same interaction with Lys 228 due to the different position of the nitrogen atom.

**Figure 3:**
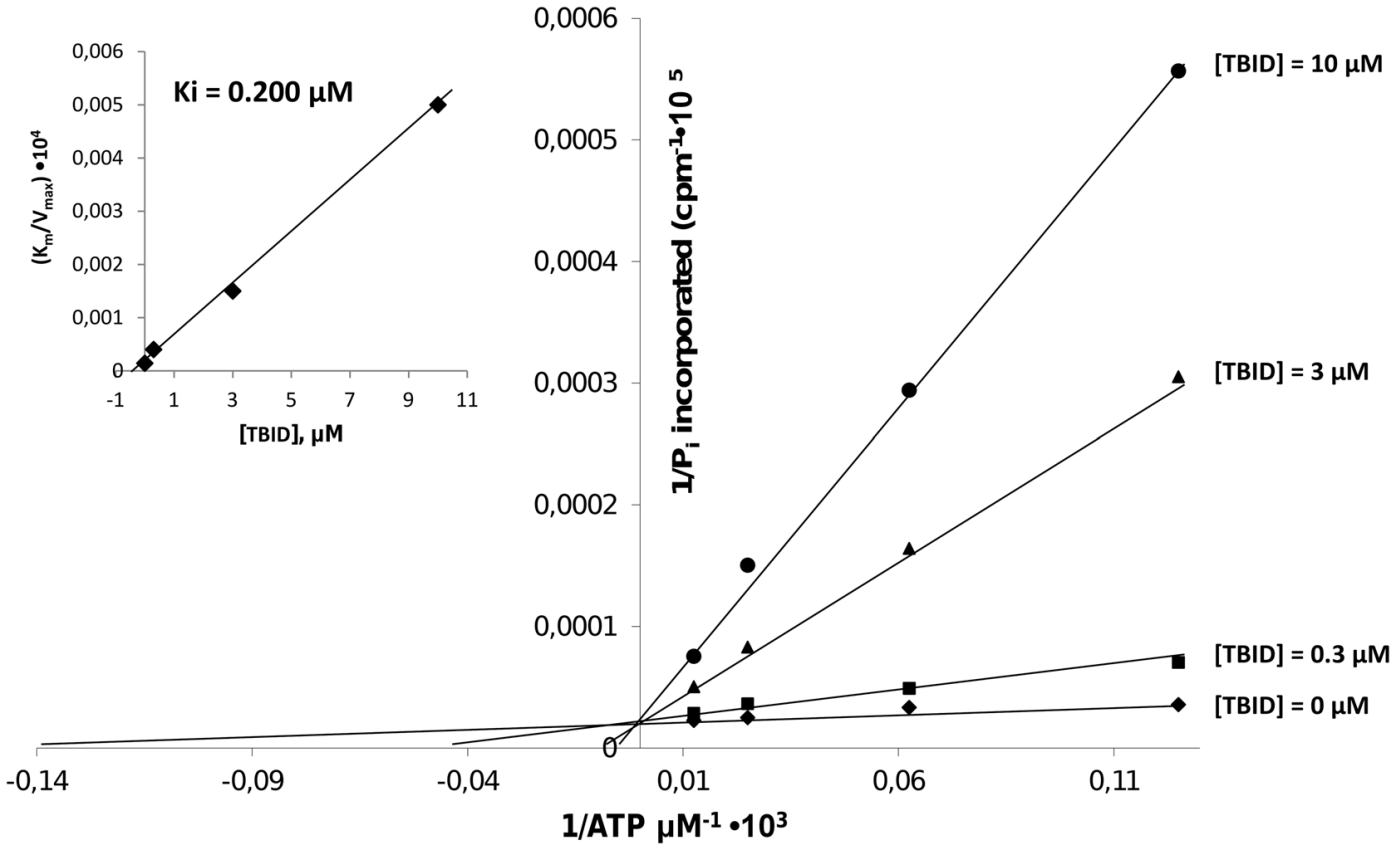
Kinetic analysis of HIPK2 inhibition by TBID. Inhibition of HIPK2 by TBID is competitive with respect to the phosphodonor substrate ATP. Kinetics were performed as described in Materials and Method either in the absence or in the presence of the indicated TBID concentrations. The data represent means of triplicate experiments with SEM never exceeding 15%.

As expected from modeling, the kinetics reported in Figure 3 show that inhibition of HIPK2 by TBID is competitive with respect to ATP. From these experiments a Ki value of 200 nM was calculated.

### 2. Selectivity of TBID

**Figure 4:**
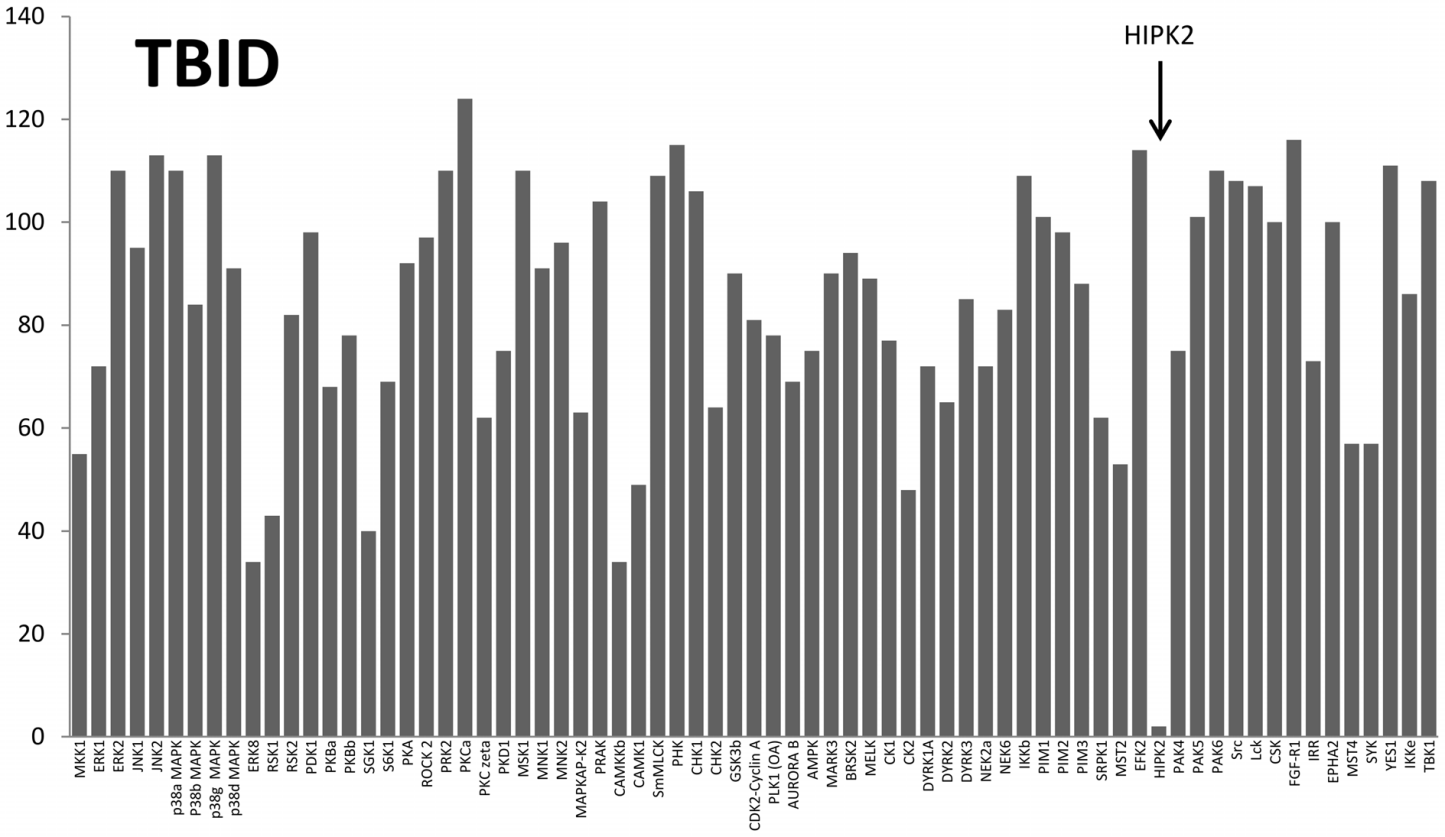
Selectivity profile of TBID (10 µM) against 70 kinases panel. Inhibition assays were performed with 10 µM TBID under conditions described or referenced in the Experimental section. Residual activity expressed as per cent of activity in the absence of TBID is shown.

The selectivity of the newly developed HIPK2 inhibitor TBID was firstly tested at 10 µM concentration on a panel of 76 protein kinases. As shown in Figure 4 the activity of HIPK2 was entirely suppressed while none of the other protein kinases underwent a similar inhibition, the residual activity of the second (BTK) and third (CAMKKb) most inhibited kinases being 29% and 34%, respectively. To note in particular the modest inhibition of those kinases which generally tend to be susceptible to CK2 inhibitors, notably CK2 itself (48% residual activity), DYRK1A (72% residual activity) and PIM1 (entirely unaffected).

To gain more information about the selectivity of TBID the compound was profiled at 1 instead of 10 µM concentration on a larger panel of 125 protein kinases, implemented with other members of the HIPK sub-family and many protein tyrosine kinases which were scarcely represented in the smaller panel. The data, shown in File S1, corroborate the concept that HIPK-2 is the kinase most susceptible to TBID (31% residual activity). HIPK1 and HIPK3 however are also significantly inhibited with residual activities of 39% and 53%, respectively. In contrast none of the protein tyrosine kinases tested is appreciably affected by TBID with the only possible exception of IGF-IR (68% residual activity). This together with CAMK1 and CAMKKb are the only kinases inhibited more than 20% a part from the HIPKs.

**Figure 5:**
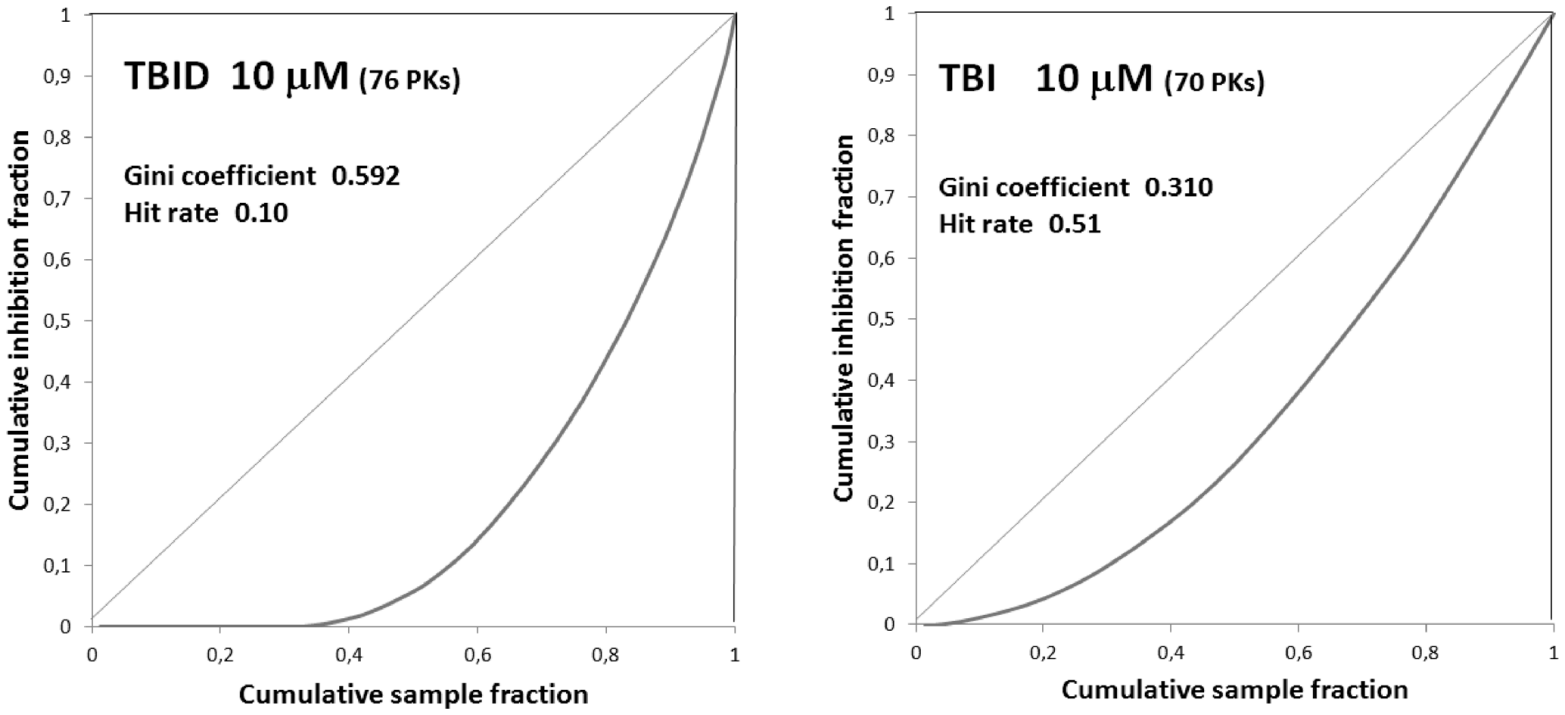
Lorenz curves, Gini coefficients and hit rates for TBID and TBI. For details see experimental section and [25]. Lorenz curves were drawn from the selectivity profile of Figure 4 and from analogous data published in ref. [19] for TBID and TBI, respectively.

Collectively taken these data denote TBID as a very selective inhibitor of HIPKs in general and HIPK2 in particular, and they highlight the striking superiority of this new compound over both TBI and SB203580. To note that in our hands SB203580 is not appreciably affecting HIPK2 activity up to 40 µM concentration (Table 1) consistent with previous reports [9], [13]. In contrast the IC_50_ values with TBI (1 µM) was only slightly higher than that with TBID, the latter however being much more selective as also highlighted by the observation that the number of kinases inhibited >90% by either 10 µM TBID or TBI in the same panel is 1(HIPK2, see Figure 4) and 10 (see ref. [20]) respectively. From the selectivity data of Figure 4 it was possible to draw a Lorenz curve (Figure 5) allowing to calculate a Gini coefficient (0.592) whose value denotes a remarkable selectivity, especially if compared to that of TBI (0.310). The difference in selectivity between TBID and TBI is also striking if their hit rates (0.10 vs 0.55) are compared.

### 3. Cell Permeability of TBID

**Figure 6:**
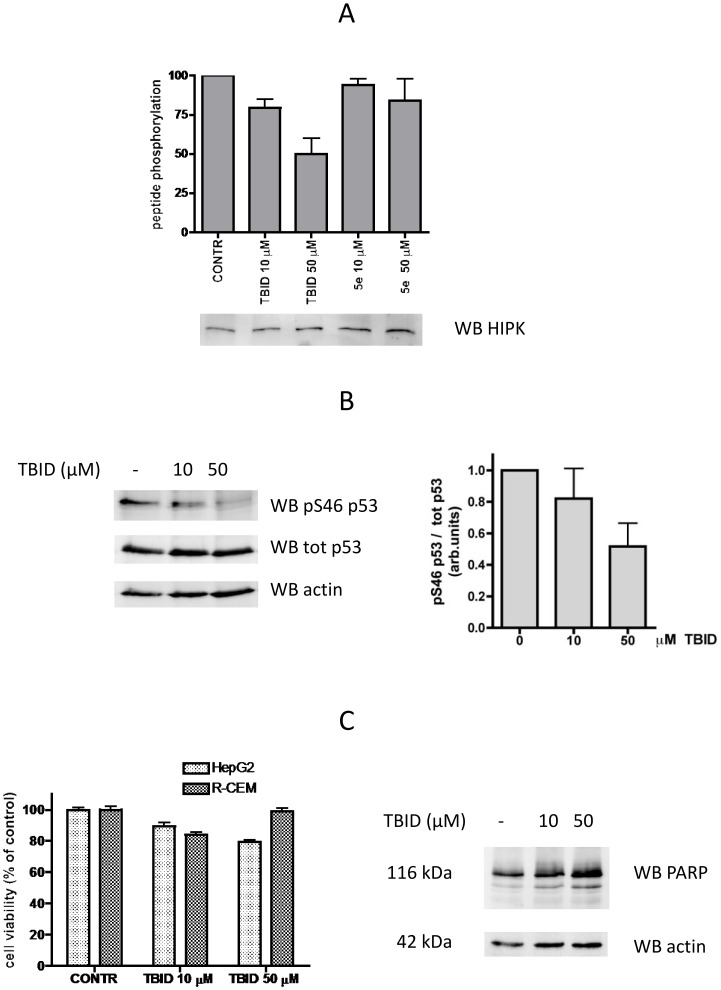
Cell treatment with TBID inhibits endogenous HIPK. A. HIPK2 was immunoprecipitated from lysates of HepG2 cells treated with different concentrations of TBID or the inactive analog **5e**, as indicated; HIPK2 activity was measure towards the specific peptide substrate, as detailed in Materials and Methods. The amount of immunoprecipitated HIPK2 is shown by WB. B. CEM cells were treated as indicated, then 10 µg of cell lysate proteins were analysed by WB with an antibody against pS46 of p53, anti-total p53, or anti-actin, as loading control. A representative experiment is shown on the left, while a histogram is presented on the right where Sp46 p53 of three separate experiments has been quantified, normalized to the p53 total level, and reported as means±SEM. C. Cell viability was assessed by the method of MTT, treating HepG2 or CEM cells as indicated (left panel) or by evaluating the amount of the caspase substrate PARP (HepG2 cells, right panel), whose reduction would denote apoptosis. The recognized band corresponds to the full length PARP protein of 116 kDa.

Dealing with protein kinase inhibitors, a crucial issue is their cell permeability which is essential to make these reagents useful for in vivo studies. Cell permeability of TBID was firstly assessed by treating HepG2 cells with increasing concentrations of either TBID or its very close analog **5e** almost devoid of inhibitory efficacy (see Table 1 and Figure 1) and measuring HIPK2 activity in the cell lysate : HIPK2 was immunoprecipitated and then assayed for its activity using a specific peptide substrate. As shown in Figure 6A endogenous HIPK2 activity is reduced in a dose dependent manner upon cell treatment with TBID, but not with its inactive analog **5e**, providing the evidence that TBID is cell permeant. Incidentally this outcome places TBID in that category of protein kinase inhibitors whose efficacy persists after the kinase has been isolated from the treated cells. Such a behaviour is typical of many CK2 inhibitors [30], [34], [35], TBB and TBI included, but it has also been reported in the case of other kinases, e.g. PIM-1 [36]. The molecular features underlying persistent inhibition, suggestive of a very low Koff rate, are presently unclear, but it is plausible to assume that these compounds, once entrapped in the hydrophobic pocket of the kinase (see also Figure 2), undergo a thermodynamic advantage, hindering their release into the surrounding aqueous medium. We also considered the possibility that intracellular TBID could irreversibly inactivate HIPK-2 by preventing the phosphorylation of its up-regulatory tyrosine, an event occurring only during translation [37], [38]. In our cell model, however, we couldn’t detect any phospho-Tyr signal in HIPK-2 immunoprecipitated from either untreated or treated cells (not shown).

To reinforce the view that endogenous HIPK-2 is inhibited upon cell treatment with TBID, advantage has been also taken of p53 Ser46, a known target of the kinase [39]. As shown in Figure 6B, TBID treatment markedly reduces the phosphorylation level of this residue, without affecting the amount of p53, under conditions devoid of cell toxicity (Figure 6C). To note that, although p53 Ser46 is not targeted exclusively by HIPK2, other putative phosphorylating agents of this residue, notably DYRK2 [40] and PKC [41], are nearly unaffected by the inhibitor under conditions where HIPK2 is >70% inhibited (see File S1). This observation, in conjunction with the similar dose dependency of HIPK2 activity inhibition and decrease of p53 Ser46 phosphorylation (compare the histograms of panels A and B in Figure 6), support the view that the reduction of p53 Ser46 phosphorylation is mainly due to HIPK2 inhibition. It should be noted in this connection that the concentration required for half maximal inhibition is two orders of magnitude higher in cells than it is in vitro (50 µM vs 0.33 µM). This is not unusual among protein kinase inhibitors [42] as exemplified elsewhere [30], [35], [43] and may be accounted for by massive sequestration of lipophilic compounds to cellular structures and to the fact that ATP competitive inhibitors (such as TBID) have to cope with a very high ATP concentration (in the mM range) within the cell.

Collectively taken, the data presented fill a gap in the field of signal transduction mediated by protein phosphorylation by making available for the first time a specific and cell permeable inhibitor for HIPK2, a protein kinase whose emerging role as regulator of cell growth and apoptosis in various tissues and whose implication in the mode of action of chemotherapeutic agents is rising remarkable interest. The only compound used so far as an HIPK2 inhibitor in fact (SB203580) was developed to inhibit different classes of protein kinases and its efficacy to inhibit HIPK2 activity is questionable, as clearly shown here and elsewhere [9], [13]. On the other hand a number of compounds able to drastically inhibit both protein kinase CK2 and HIPK2 [17], [19] display a wide promiscuity, which hampers their usage as selective HIPK2 inhibitors. In contrast, the compound whose synthesis and characterization are described here, TBID, displays a good efficacy and a remarkable selectivity towards the members of the HIPK family, with special reference to HIPK2, as shown both by profiling it on large panels of kinases and by molecular modelling, accounting for its ATP competitive mode of action. These properties, in conjunction with ability to permeate cells, as judged from inhibition of endogenous HIPK2, make TBID the first choice and for the time being the only pharmacological tool to down regulate cellular HIPK2, with the caveat that the concentrations of the compound effective in cells are much higher than the IC_50_ values calculated in vitro.

## Supporting Information

File S1 **Table S1,** Selectivity profiles of TBID on a 125 kinase panel. These are expressed in % activity of the enzyme. **Figure S1,** Synthesis of 4,5,6,7-tetrabromo-2-(1*H*-imidazol-2-yl)isoindoline-1,3-diones. Reagents and conditions: (i) acetic acid, reflux, 1–3 h, 11–64%. For synthesis of 5a, instead of a free heterocyclic base the 2-aminoimidazolium sulfate was used in the presence of DBU.
(DOC)
