# Supplementary material for: Synthesis and Properties of a Selective Inhibitor of Homeodomain–Interacting Protein Kinase 2 (HIPK2)
Source: PLoS One. 2014 Feb 24;9(2):e89176. doi: 10.1371/journal.pone.0089176 (PMC3933419; doi:10.1371/journal.pone.0089176)
Supplement: File S1 — Table S1, Selectivity profiles of TBID on a 125 kinase panel. These are expressed in % activity of the enzyme. Figure S1, Synthesis of 4,5,6,7-tetrabromo-2-(1H-imidazol-2-yl)isoindoline-1,3-diones. Reagents and conditions: (i) acetic acid, reflux, 1–3 h, 11–64%. For synthesis of 5a, instead of a free heterocyclic base the 2-aminoimidazolium sulfate was used in the presence of DBU. (DOC) [file pone.0089176.s001.doc]

**Synthesis and properties of a selective inhibitor of Homeodomain–Interacting Protein Kinase 2 (HIPK2)**

Giorgio Cozza^1^, Sofia Zanin^1^, Renate Determann^2^, Maria Ruzzene^1, 3^, Conrad Kunick^2^, Lorenzo A. Pinna^1, 3*^

^1^ Department of Biomedical Sciences, University of Padova, and CNR Institute of Neurosciences, Padova, Italy

^2^ Technische Universität Braunschweig, Institut für Medizinische und Pharmazeutische Chemie, Braunschweig, Germany

^3^ Venetian Institute of Molecular Medicine (VIMM), Padova, Italy

^*^Correspondence: Lorenzo A. Pinna Department of Biomedical Sciences, University of Padova, Viale G. Colombo 3 35131 Padova, Italy tel. +39 049 8276108, Fax. +39 049 8073310, email: [lorenzo.pinna@unipd.it](mailto:lorenzo.pinna@unipd.it)

**Table S1** Selectivity profiles of TBID on a 125 kinase panel. These are expressed in % activity of the enzyme

| KINASE | TBID 1 μM |
| --- | --- |
| HIPK2 | **31** |
| HIPK1 | **39** |
| HIPK3 | **53** |
| IGF-1R | **68** |
| CAMKKb | **75** |
| SRPK1 | **77** |
| TAK1 | **78** |
| CAMK1 | **78** |
| NUAK1 | **81** |
| IRAK1 | **82** |
| Src | **83** |
| PLK1 | **83** |
| MLK1 | **83** |
| BTK | **84** |
| IKKe | **85** |
| CK2 | **87** |
| AMPK | **87** |
| p38a MAPK | **88** |
| EPH-A2 | **89** |
| CSK | **89** |
| NEK2a | **89** |
| MSK1 | **89** |
| MKK6 | **90** |
| MST2 | **90** |
| ZAP70 | **91** |
| ERK2 | **91** |
| MINK1 | **91** |
| HER4 | **91** |
| TTK | **92** |
| DAPK1 | **92** |
| RSK1 | **93** |
| MPSK1 | **93** |
| DYRK2 | **94** |
| S6K1 | **94** |
| GSK3b | **94** |
| ERK8 | **95** |
| PRAK | **95** |
| MARK4 | **95** |
| EPH-B1 | **96** |
| PRK2 | **96** |
| MKK2 | **96** |
| PAK2 | **97** |
| MLK3 | **97** |
| PDK1 | **97** |
| CDK2-Cyclin A | **98** |
| MAPKAP-K3 | **98** |
| SGK1 | **98** |
| ERK1 | **98** |
| PKD1 | **98** |
| PAK4 | **99** |
| BRSK2 | **99** |
| IKKb | **99** |
| ABL | **99** |
| TrkA | **100** |
| EPH-A4 | **100** |
| IRR | **100** |
| GCK | **100** |
| MARK2 | **100** |
| CHK2 | **100** |
| Aurora A | **101** |
| JNK3 | **101** |
| CLK2 | **101** |
| IR | **101** |
| IRAK4 | **101** |
| PIM3 | **101** |
| PIM2 | **101** |
| DYRK3 | **101** |
| RSK2 | **101** |
| p38g MAPK | **101** |
| PKCa | **102** |
| LKB1 | **102** |
| CHK1 | **102** |
| RIPK2 | **102** |
| MARK1 | **102** |
| PAK6 | **102** |
| OSR1 | **103** |
| TESK1 | **103** |
| EPH-B3 | **103** |
| SmMLCK | **103** |
| BRK | **103** |
| FGF-R1 | **104** |
| PKBb | **104** |
| PKBa | **104** |
| PKCz | **104** |
| PIM1 | **104** |
| DYRK1A | **104** |
| PKA | **104** |
| PAK5 | **105** |
| CK1 | **105** |
| NEK6 | **105** |
| MNK1 | **106** |
| Aurora B | **106** |
| p38d MAPK | **106** |
| MEKK1 | **106** |
| JNK1 | **106** |
| EIF2AK3 | **107** |
| TLK1 | **107** |
| TBK1 | **108** |
| MST4 | **108** |
| EPH-B2 | **108** |
| SYK | **108** |
| Lck | **109** |
| TSSK1 | **109** |
| BRSK1 | **110** |
| MELK | **110** |
| JNK2 | **110** |
| YES1 | **110** |
| TAO1 | **110** |
| p38b MAPK | **111** |
| EF2K | **111** |
| STK33 | **111** |
| TTBK1 | **113** |
| MNK2 | **113** |
| EPH-B4 | **114** |
| JAK2 | **114** |
| MKK1 | **115** |
| MKK1 | **115** |
| TIE2 | **115** |
| VEG-FR | **115** |
| MARK3 | **115** |
| PHK | **116** |
| PKCγ | **117** |
| ROCK 2 | **117** |
| MAPKAP-K2 | **119** |
| ASK1 | **126** |

**Chemistry: General**

**Figure S1**: Synthesis of 4,5,6,7-tetrabromo-2-(1*H*-imidazol-2-yl)isoindoline-1,3-diones **.**

Reagents and conditions: (i) acetic acid, reflux, 1-3 h, 11-64%. For synthesis of **5a**, instead of a free heterocyclic base the 2-aminoimidazolium sulfate was used in the presence of DBU.

Starting materials **3** and **4** were purchased from commercial suppliers and were used without further purification.

4,5,6,7-Tetrabromo-2-(1*H*-imidazol-2-yl)isoindoline-1,3-dione (**5a**)

2-Aminoimidazolium sulfate (132 mg, 0.50 mmol) and DBU (23 µL, 1.5 mmol) were stirred in acetic acid (10 mL) for 5 min. Subsequently, tetrabromophthalic anhydride (1.00 mmol, 464 mg) was added in portions and the mixture was refluxed for 4 h. A precipitate was formed after cooling to room temperature, which was filtered off by suction and washed successively with water and petrol ether. Purification was carried out by continuous extraction with ethyl acetate by means of a Soxhlet apparatus. Subsequent crystallization form acetone to yielded 122 mg (23%) of a yellow powder, . mp. > 360 °C; IR (KBr) 3434 cm^-1^ (NH), 1724 cm^-1^ (C=O); ^1^H NMR (DMSO-d_6_, 600.1 MHz) δ (ppm) = 7.04 (s, 1H, ArH), 7.30 (s, 1H, ArH), 12.35 (s, 1H, NH); ^13^C NMR (DMSO-d_6_, 150.9 MHz) δ (ppm) = 118.1, 127.7 (tert. C); 121.2 (2C), 130.4 (2C), 131.2 (2C), 137.3, 162.2 (2C) (quat. C); C_11_H_3_Br_4_N_3_O_2_ (528.78); calcd. C 24.99, H 0.57, N 7.95; found C 25.32, H 0.60, N 8.24; HPLC: 98.2 % at 254 nm and 99.0 % at 280 nm, t_N_ = 2.93 min, t_M_ = 1.06 min (ACN/buffer (pH = 2.6); 45:55), λ_max_: 246 nm and 343 nm.

*General Procedure for the Synthesis of 2-aryl substituted 4,5,6,7-Tetrabromoisoindoline-1,3-diones 5b-i*

Tetrabromophthalic anhydride (**3**; 464 mg, 1.00 mmol) and a suitable aromatic amine (**4**; 1.00 mmol) are refluxed in acetic acid (10 mL) for 1-3 h. The reaction is monitored by TLC. After cooling to room temperature a solid precipitates, which is filtered off by suction and washed successively with water and petrol ether. The material is purified as indicated in the synthesis procedures for distinct compounds.

4,5,6,7-Tetrabromo-2-(thiazol-2-yl)isoindoline-1,3-dione (**5b**)

Prepared following the general procedure. Reaction time: 1 h. Crystallization from acetone yielded 61 mg (11%) colorless powder; mp. 228-232 °C (Rice et al. 1968, J. Med. Chem 11: 225-227 °C); IR (KBr) 1784 cm^-1^ and 1728 cm^-1^ (C=O); ^1^H NMR (DMSO-d_6_, 600.1 MHz) δ (ppm) = 7.86 (d, 1H, *J* = 3.6, thiazol-H), 7.88 (d, 1H, *J* = 3.6, thiazol-H); ^13^C NMR (DMSO-d_6_, 150.9 MHz) δ (ppm) = 120.8, 140.0 (tert. C); 121.1 (2C), 130.7 (2C), 137.0 (2C), 151.0, 161.0 (2C) (quat. C); C_11_H_2_Br_4_N_2_O_2_S (545.83); calcd. C 24.21, H 0.37, N 5.13; found C 24.33, H 0.20, N 4.82; HPLC: 98.4 % at 254 nm and 98.3 % at 280 nm, t_N_ = 3.97 min, t_M_ = 1.06 min (ACN/H_2_O; 60:40), λ_max_: 250 nm and 341 nm.

4,5,6,7-Tetrabromo-2-(1,3,4-thiadiazol-2-yl)isoindoline-1,3-dione (**5c**)

Prepared following the general procedure. Reaction time: 1 h. Crystallization from acetone yielded 61 mg (11%) colorless powder; mp. 310-311 °C; IR (KBr) 1784 cm^-1^ and 1734 cm^-1^ (C=O); ^1^H NMR (DMSO-d_6_, 600.1 MHz) δ (ppm) = 9.69 (s, 1H, thiadiazol-H); ^13^C NMR (DMSO-d_6_, 150.9 MHz) δ (ppm) = 154.15 (tert. C); 121.4 (2C), 130.7 (2C), 137.4 (2C), 153.5, 160.4 (2C) (quat. C); C_10_HBr_4_N_3_O_2_S (546.82); calcd. C 21.97, H 0.18, N 7.68; found C 22.14, H 0.05, N 7.61; HPLC: 96.6 % at 254 nm and 96.0 % at 280 nm, t_N_ = 4.27 min, t_M_ = 1.03 min (ACN/H_2_O; 50:50), λ_max_: 261 nm and 345 nm.

4,5,6,7-Tetrabromo-2-(1,2,4-thiadiazol-5-yl)isoindoline-1,3-dione (**5d**)

Prepared following the general procedure. Reaction time: 1 h. Purification by refluxing in acetone yielded 276 mg (51%) colorless powder; mp. 325-326 °C; IR (KBr) 1733 cm^-1^ (C=O); ^1^H NMR (DMSO-d_6_, 600.1 MHz) δ (ppm) = 8.84 (s, 1H, thiazol-H); ^13^C NMR (DMSO-d_6_, 150.9 MHz) δ (ppm) = 159.9 (tert. C); 121.7 (2C), 130.7 (2C), 137.8 (2C), 159.7, 169.9 (2C) (quat. C); C_10_HBr_4_N_3_O_2_S (546.82); calcd. C 21.97, H 0.18, N 7.68; found C 22.19, H 0.18, N 7.78; HPLC: 90.0 % at 254 nm and 93.1 % at 280 nm, t_N_ = 3.40 min, t_M_ = 1.06 min (ACN/H_2_O; 60:40), λ_max_: 268 nm and 346 nm.

4,5,6,7-Tetrabromo-2-(1*H*-pyrazol-5-yl)isoindoline-1,3-dione (**5e**)

Prepared following the general procedure. Reaction time: 1 h. Crystallization from acetone yielded 62 mg (12%) colorless powder; mp. 333-334 °C (dec.); IR (KBr) 3331 cm^-1^(NH), 3152 cm^-1^ (CH ar.), 1780 cm^-1^ and 1724 cm^-1^ (C=O); ^1^H NMR (DMSO-d_6_, 600.1 MHz) δ (ppm) = 6.36 (d, 1H, *J* = 2.0 Hz, pyrazol-H), 7.89 (d, 1H, *J* = 2.0 Hz, pyrazol-H), 13.18 (s,1H, NH); ^13^C NMR (DMSO-d6, 150.9 MHz) δ (ppm) = 102.5, 130.0 (tert. C); 120.8 (2C), 130.9 (2C), 136.6 (2C), 139.4, 162.5 (2C) (quat. C); C_11_H_3_Br_4_N_3_O_2_ (528.78); calcd. C 24.99, H 0.57, N 7.95; found C 24.82, H 0.64, N 7.54; HPLC: 99.6 % at 254 nm and 99.0 % at 280 nm, t_N_ = 4.00 min, t_M_ = 1.06 min (ACN/H2O; 50:50), λ_max_: 247 nm and 340 nm.

4,5,6,7-Tetrabromo-2-(3-methyl-1*H*-pyrazol-5-yl)isoindoline-1,3-dione (**5f**)

Prepared following the general procedure. Reaction time: 1 h. Crystallization from acetone yielded 146 mg (27%) colorless powder; mp. 331-332 °C; IR (KBr) 3443 cm^-1^, 3255 cm^-1^ (NH), 3154 cm^-1^ (CH arom.), 2922 cm^-1^ (CH aliph.), 1778 cm^-1^ and 1730 cm^-1^ (C=O); ^1^H NMR (DMSO-d_6_, 600.1 MHz) δ (ppm) = 2.29 (s, 3H, CH_3_), 6.09-6.10 (dd, 1H, *J* = 1.3/0.8, pyrazol-H), 12.85 (br s, 1H, NH); ^13^C NMR (DMSO-d_6_, 150.9 MHz) δ (ppm) = 10.6 (CH_3_); 101.5 (tert. C); 120.8 (2C), 130.8 (2C), 136.6 (2C), 139.5, 139.7, 162.5 (2C) (quat. C); C_12_H_5_Br_4_N_3_O_2_ (542.80); calcd. C 26.55, H 0.93, N 7.74; found C 26.78, H 0.96, N 7.55; HPLC: 99.2 % at 254 nm and 99.4% at 280 nm, t_N_ = 5.12 min, t_M_ = 1.06 min (ACN/H_2_O; 50:50), λ_max_: 246 nm and 340 nm.

4,5,6,7-Tetrabromo-2-(1*H*-1,2,4-triazol-5-yl)isoindoline-1,3-dione (**5g**)

Prepared following the general procedure. Reaction time: 1 h. Purification by refluxing in acetone yielded 339 mg (64%) colorless powder; mp. >360 °C; IR (KBr) 3430 cm^-1^(NH), 2853 cm^-1^ (CH arom.), 1784 cm^-1^ and 1736 cm^-1^ (C=O); ^1^H NMR (DMSO-d_6_, 600.1 MHz) δ (ppm) = 8.79 (s, 1H, triazol-H), 14.60 (s, 1H, NH); ^13^C NMR (DMSO-d_6_, 150.9 MHz) δ (ppm) = 145.3 (tert. C); 121.2 (2C), 130.7 (2C), 137.0 (2C), 149.6, 162.1 (2C) (quat. C); C_10_H_2_Br_4_N_4_O_2_ (529.77); calcd. C 22.67, H 0.38, N 10.58; found C 22.71, H 0.22, N 10.15; HPLC: 98.7 % at 254 nm and 97.0 % at 280 nm, t_N_ = 5.45 min, t_M_ = 1.06 min (ACN/H_2_O; 40:60), λ_max_: 249 nm and 343 nm.

4,5,6,7-Tetrabromo-2-(isoxazol-3-yl)isoindoline-1,3-dione (**5h**)

Prepared following the general procedure. Reaction time: 1 h. Purification by refluxing in acetone yielded 286 mg (54%) colorless powder. mp. 290-293 °C; IR (KBr) 1741 cm^-1^ (C=O); ^1^H NMR (DMSO-d_6_, 600.1 MHz) δ (ppm) = 6.90 (d, 1H, *J* = 1.8 Hz, isoxazol-H), 9.15 (d, 1H, *J* = 1.8 Hz, isoxazol-H); ^13^C NMR (DMSO-d_6_, 150.9 MHz) δ (ppm) = 102.2, 161.8 (tert. C); 121.1 (2C), 130.9 (2C), 137.0 (2C), 152.5, 160.9 (2C) (quat. C); C_11_H_2_Br_4_N_2_O_3_ (529.77); calcd. C 24.94, H 0.38, N 5.29; found C 25.02, H 0.22, N 4.96; HPLC: 99.2 % at 254 nm and 95.0 % at 280 nm, t_N_ = 3.37 min, t_M_ = 1.03 min (ACN/H_2_O; 60:40), λ_max_: 252 nm and 343 nm.

4,5,6,7-Tetrabromo-2-(pyrazin-2-yl)isoindoline-1,3-dione (**5i**)

Prepared following the general procedure. Reaction time: 1 h. Purification by refluxing in acetone yielded 61 mg (11%) of a colorless powder. mp.: 272-275 °C; IR (KBr) 1716 cm^-1^ (C=O); ^1^H NMR (DMSO-d_6_, 600.1 MHz) δ (ppm) = 8.81-8.83 (m, 3H, pyrazine-H); ^13^C NMR (DMSO-d_6_, 150.9 MHz) δ (ppm) = 143.6, 144.2, 145.0 (tert. C); 121.1 (2C), 130.8 (2C), 137.0 (2C), 142.2, 162.1 (2C) (quat. C); C_12_H_3_Br_4_N_3_O_2_ (540.79); calcd. C 26.65, H 0.56, N 7.77; found C 26.68, H 0.38, N 7.58; HPLC: 98.5 % at 254 nm and 96.0 % at 280 nm, t_N_ = 5.93 min, t_M_ = 1.06 min (ACN/buffer (pH = 2.6); 50:50), λ_max_: 252 nm and 343 nm.
